# Biomechanical properties of the aortic root are distinct from those of the ascending aorta in both normal and aneurysmal states

**DOI:** 10.1016/j.xjon.2023.08.015

**Published:** 2023-09-04

**Authors:** Jennifer C.-Y. Chung, Daniella Eliathamby, Hijun Seo, Chun-Po Fan, Rifat Islam, Karamvir Deol, Craig A. Simmons, Maral Ouzounian

**Affiliations:** a Division of Cardiovascular Surgery, Peter Munk Cardiac Centre, University Health Network, Toronto General Hospital, Toronto, Ontario, Canada; b Department of Surgery, University of Toronto, Toronto, Ontario, Canada; c Institute of Biomedical Engineering, University of Toronto, Toronto, Ontario, Canada; d Translational Biology & Engineering Program, Ted Rogers Centre for Heart Research, The Hospital for Sick Children, Toronto, Ontario, Canada; e Rogers Computational Program, Peter Munk Cardiac Centre, University Health Network, Toronto, Ontario, Canada; f Department of Mechanical & Industrial Engineering, University of Toronto, Toronto, Ontario, Canada

**Keywords:** aortic aneurysm, aortic biomechanics, aortic dissection, aortic root, ascending aorta

## Abstract

**Background:**

Although aneurysms of the ascending aorta and the aortic root are treated similarly in clinical guidelines, how biomechanical properties differ between these 2 segments of aorta is poorly defined.

**Methods:**

Biomechanical testing was performed on tissue collected from the aortic root (normal = 11, aneurysm = 51) and the ascending aorta (normal = 21, aneurysm = 76). Energy loss, tangent modulus of elasticity, and delamination strength were evaluated. These biomechanical properties were then compared between (1) normal ascending and normal root tissue, (2) normal and aneurysmal root tissue, (3) normal and aneurysmal ascending tissue, and (4) aneurysmal root and aneurysmal ascending tissue. Propensity score matching was performed to further compare aneurysmal root and aneurysmal ascending aortic tissue. Clinical and biomechanical variables associated with decreased delamination strength in the aortic root were evaluated.

**Results:**

The normal aortic root demonstrated greater viscoelastic behavior (energy loss 0.08 [0.06, 0.10] vs 0.05 [0.04, 0.06], *P* = .008), and greater resistance against delamination (93 [58, 126] mN/mm vs 54 [40, 63] mN/mm, *P* = .05) compared with the ascending aorta. Delamination strength was significantly reduced in aneurysms in both the root and the ascending aorta compared with their normal states. Aneurysms of the aortic root matched to the ascending aortic aneurysms in terms of baseline characteristics including size, were characterized by a larger decrease in delamination strength from baseline (Δ −59 mN/mm vs Δ −24 mN/mm). Aging (*P* = .003) and the presence of hypertension (*P* = .02) were associated with weakening of the aortic root, while diameter did not have this association (*P* = .29).

**Conclusions:**

The normal aortic root was found to have distinct biomechanical properties compared with the ascending aorta. When aneurysms form in the aortic root, there is less strength against delamination, without other biomechanical changes such as increased energy loss observed in aneurysmal ascending aortas. Age and hypertension were associated decreased aortic wall strength in the aortic root, whereas diameter had no such association.

## Graphical abstract

**Figure:**
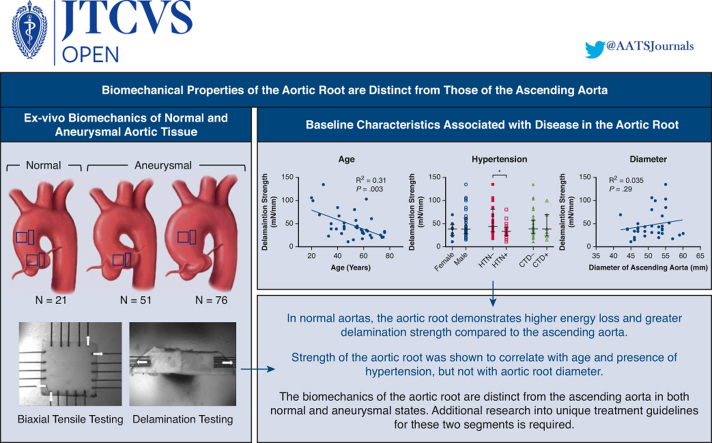

**Figure:**
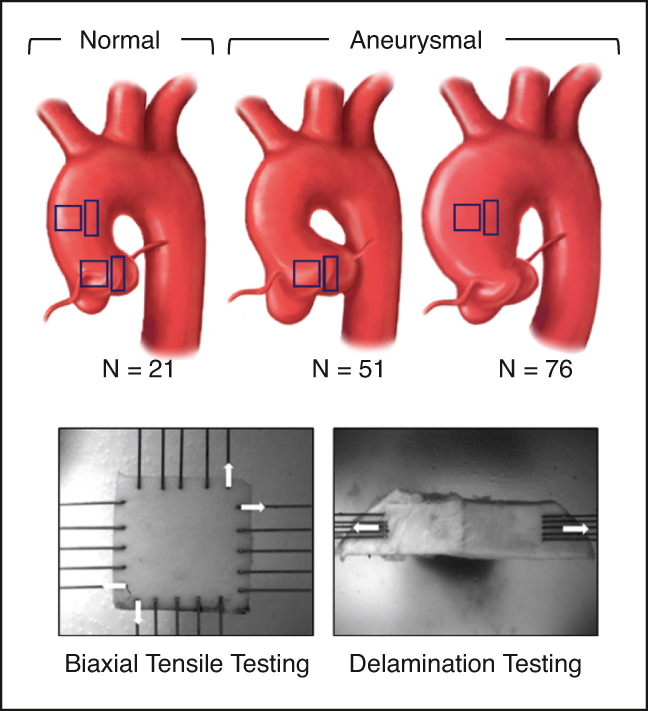
Biomechanical comparison of ascending aortic and aortic root tissue.

**Central Message**

The biomechanics of the aortic root are distinct from the ascending aorta in both normal and aneurysmal states. Additional research into unique treatment guidelines for these 2 segments is required.

**Perspective**

Current management of aortic disease between the ascending aorta and aortic root is similar. However, when comparing the material properties between these 2 regions, distinct differences in how these 2 segments of the aorta behave in both the normal and aneurysmal states were observed. Additional research into unique guidelines for disease management in these 2 segments is required.

The aortic root differs from the ascending aorta in terms of structure and function. The sinuses of Valsalva, which originate from the cusp insertion line to the sinotubular junction, are shaped differently from the tubular ascending aorta. Hydrodynamic studies show that the unique shape of the aortic root minimizes stress and deformation on the aortic cusps,^1^ whereas the main function of the ascending aorta is as a conduit and a capacitor.^2^ Despite these differences, aortic tissue in both of these anatomical locations may become aneurysmal, which increases their risk of aortic dissection. American and European guidelines on the management of aortic disease do not distinguish between the 2 locations with respect to their recommendations on timing of surgical intervention.^3^^,^^4^

Studies of aortic biomechanics by our group and others have focused primarily on the ascending aorta.^5^, ^6^, ^7^, ^8^ These studies have shed light on the normal biomechanical properties of the ascending aorta and how they change with disease. A complex interplay between how various biomechanical properties are disproportionately altered depending on patient characteristics including age and valve type have been observed.^9^, ^10^, ^11^ Multiple studies describe the relationship between biomechanical properties and the underlying microstructure.^8^^,^^12^ More recently, biomechanical properties have been linked to wall shear stress and hemodynamic alterations that occur at the wall in studies by our group and others.^13^, ^14^, ^15^ Taken together, the last few decades have increased our understanding of how and why the aortic wall fails, with the aim of prediction and prevention of aortic catastrophe in patients.

Despite this progress, there is a great paucity of data on the biomechanical properties of the aortic root. Using well-developed methodology for the study of the ascending aorta as a starting point, we set out to describe the biomechanical properties of normal and aneurysmal human aortic roots, as well as to carry out comparative studies with normal and aneurysmal human ascending aortas.

## Methods

This study was approved by the research ethics board of the University Health Network, Toronto, Canada (research ethics board #16-6285, July 27, 2017). All participants provided written informed consent. Isolated ascending aortic samples from 76 patients and isolated aortic root samples from 51 patients undergoing elective aortic surgery for aneurysmal disease were collected between July 2017 and February 2022. Patients with concomitant ascending and root aneurysms were excluded from this study (n = 18). Normal ascending aortas (N = 21) and normal aortic roots (N = 11) were collected from transplant donors including those that were not used for heart transplantation. Clinical data were prospectively collected.

### Mechanical Testing

#### Sample preparation

**Figure 1:**
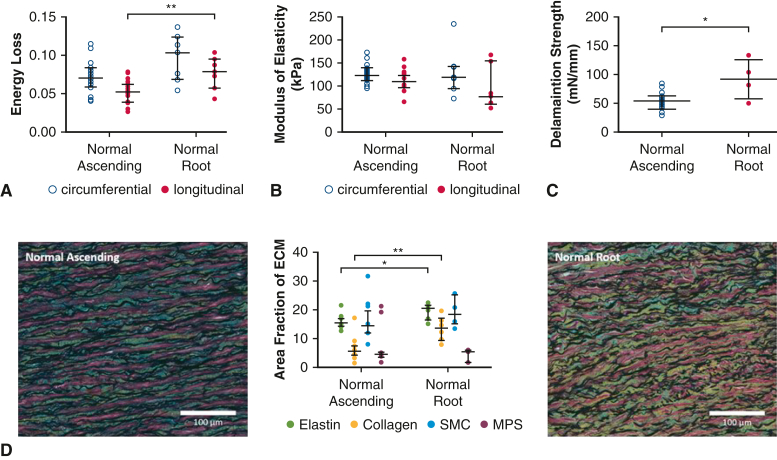
Biomechanical comparison between normal ascending aortas and normal aortic roots. Greater energy loss is observed in normal aortic roots compared with normal ascending aortas in the longitudinal axis of tissue (A). No significant difference in modulus of elasticity between normal aortic roots and normal ascending aortas was observed (B). Delamination strength was significantly greater in normal aortic roots compared with normal ascending aortas (C). Histologic comparison between normal ascending aortas and normal aortic roots (D). Percentage area of both elastin and collagen were significantly greater in normal aortic roots compared with normal ascending aortas. All data are displayed as median with interquartile range. ∗*P* ≤ .05, ∗∗*P* ≤ .01. *SMC*, Smooth muscle cell; *MPS*, mucopolysaccharide.

All aortic tissues were collected from the operating room and immediately placed on ice. The tissues were then stored at 4 °C in Ringer's lactate solution and tested within 24 hours of collection. For the ascending aorta, specimens were received in the form of a complete ring, with orientation marked by the surgeon (Figure 1, *A*). A custom-made 14-mm × 14-mm cutter was used to collect square samples from the outer curvature for biaxial tensile tests. Up to 3 square samples were cut along the longitudinal axis. Adjacent to these squares, a 6-mm × 30-mm rectangular piece was cut for delamination testing (Figure 1, *B*).

For aortic root specimens, the noncoronary sinus of Valsalva was excised and the sinotubular junction marked by a clip. Then, from the belly of the sinus, a single 14-mm × 14-mm square was sampled along with a single adjacent 6-mm × 30-mm rectangular sample. Mostly we found that only one or the either was possible; therefore, for the first half of the study, we prioritized the square samples and then transitioned to the rectangular samples.

#### Biaxial tensile testing

Biaxial tensile testing was used to derive energy loss and tangent modulus of elasticity. Energy loss is a measure of the efficiency with which the aorta performs the Windkessel function. Greater values of energy loss signify a greater proportion of energy absorbed by the aorta during loading is dissipated and less of it is returned to the circulation. Modulus of elasticity measures tissue stiffness, with greater values indicating greater stiffness. Aneurysmal ascending aortic tissue tends to have greater levels of both energy loss and tangent modulus of elasticity.

Aortic thickness for biaxial tensile testing was measured using a high-magnification (12×) zoom lens (Navitar) before mechanical testing. Biaxial testing was performed as previously described.^9^ To summarize, each biaxial square sample was mounted onto a Biotester (CellScale) using tungsten rakes for displacement controlled biaxial tensile testing in 37 °C Ringer's lactate solution (Figure 1, *C*). Samples were subjected to 10 preconditioning stretch cycles followed by 3 analyzed cycles under 25% equibiaxial strain. From the generated stress–strain curve, energy loss and the tangent modulus of elasticity at 10% strain were calculated in both the longitudinal (long) and circumferential (circ) directions of loading.

#### Delamination testing

Aortic tissue that is resistant to dissection has a greater delamination strength than aortas that are prone to dissection. Delamination tests were performed on the rectangular tissue samples as previously described.^9^ Tissue width was confirmed using the built in Biotester camera and measured using ImageJ, version 1.52p (National Institutes of Health). An initial incision was made in the media and the medial layer was manually peeled from the proximal to distal end of the aorta tissue section until 14 mm of unpeeled length remained. The samples were then mounted onto the Biotester for uniaxially peeling at a constant rate of 0.01 mm/s in 37 °C Ringer lactate solution (Figure 1, *D*). Force measurements were recorded, and delamination strength was found by dividing average force by sample width.

### Imaging Analysis

Computed tomography or magnetic resonance imaging measurements was used. Image analysis was performed using Aquarius iNtuition (TeraRecon, Inc). To summarize, a semiautomatic centerline was generated through the ascending aorta starting at the left ventricular outflow tract, with manual inspection for appropriate symmetry around the centerline. Scrolling through the centerline, for aortic root measurements, maximal cusp-to-cusp as well as cusp-to-commissure diameters were recorded. Results based on cusp-to-cusp measurements are reported here, but findings were unchanged when using cusp-to-commissure measurements instead. The maximal diameter measurement for the ascending aorta was generated from built-in volume analysis tools, with manual adjustments as needed. A small proportion of patients only had echocardiography data (mostly the controls), in which case cardiologist-reported diameters were used.

### Histology

Histology preparation and staining were performed as previously described.^9^ Orientated square aortic samples were fixed in 10% formalin following biaxial tensile testing. Each sample was then paraffin-embedded and cut in 4-μm thick sections for Movat's pentachrome staining. Using an Aperio ImageScope, version 12.4.0.5043 (Leica Biosystems), 3 random locations from each digitally scanned slide of aortic media were captured at 20× magnification. Images were saved and colorimetric analysis was performed using ImageJ, version 1.52p. To summarize, the YUV color threshold of each image was manually adjusted to quantify the percent area of the following components: collagen shown as yellow, smooth muscle cell shown as red, and mucopolysaccharide shown as blue. Images were then converted to 32-bit grayscale to determine the composition percentage of elastin, shown as black.

### Statistical Analysis

All continuous variables are presented as median and interquartile range [quartile 1, quartile 3]. All categorical variables are presented as an absolute number with accompanying percentage. The 4 analyses of biomechanical properties that we carried out were normal ascending versus normal root tissue, normal versus aneurysmal root tissue, normal versus aneurysmal ascending tissue, and aneurysmal root versus aneurysmal ascending tissue. To compare continuous variables, Mann–Whitney *U* tests were used. To compare between 2 categorical variables, χ^2^ tests were used. Linear regression models were used to quantify the association of aortic biomechanics with aortic diameter and length. We used an alpha value of 0.05.

Next, each case of aortic root aneurysm was matched to a case of ascending aortic aneurysm using multivariable matching on age, diameter of the aneurysm, patient's sex, valve morphology (ie, bicuspid vs tricuspid aortic valve), and history of hypertension. Missing values in delamination strength were singly imputed before matching. Postmatching analysis was conducted to quantify the effect of aneurysm location using paired *t*-tests. We further conducted pair-analysis adjusted for residual confounding. Prism 5 (GraphPad) and R 4.03 were used.

## Results

### Comparing Normal Ascending and Root Tissue

The patients who provided normal aortic tissue were 46 [35, 55] years old and mostly male (12/21, 63%). All had normally functioning tricuspid aortic valves. The median ascending aortic diameter was 28 [27, 34] mm, and the median aortic root diameter was 30 [26-36] mm. A total of 5/21 (26%) of patients had hypertension. Of note, the normal aortic root group (n = 11) was a subset of this larger ascending control group (n = 21), and therefore no comparative statistics were performed on the baseline characteristics of these 2 groups.

In normal aortas, the aortic root demonstrated greater levels of energy loss than the ascending aorta (longitudinal energy loss: 0.08 [0.06, 0.10] vs 0.05 [0.04, 0.06], *P* = .008) and similar levels of modulus of elasticity (circumferential: *P* = .95; longitudinal: *P* = .11) (Figure 1). The delamination strength of the normal aortic root was greater than that of the ascending aorta (93 [58, 126] mN/mm vs 54 [40, 63] mN/mm, *P* = .05).

On histologic analysis, percent area of collagen was significantly increased in the aortic root relative to the ascending aorta. The percent area of elastin was also increased in the root, but the difference was less striking. Content of smooth muscle cells and mucopolysaccharides were comparable (Figure 1, *D*).

### Comparing Normal and Aneurysmal Aortic Root

Age and sex of patients with normal and aneurysmal aortic roots were similar (Table 1). As expected, the aortic diameter was larger in the aneurysm group (50 [46, 54] mm vs 30 [26, 36] mm, *P* < .0001), which also had a greater rate of aortic valve pathology (Table 1).

**Table 1:** Baseline characteristics of the patient groups

Patient characteristics	Normal root (N = 11)	Normal ascending (N = 21)	Aneurysm root (N = 51)	Aneurysmal ascending (N = 76)	*P* value	
					Normal root vs aneurysmal root	Aneurysmal root vs aneurysmal ascending
Age, y	41 [35, 54]	47 [35, 56]	53 [39,62]	67 [57, 75]	.21	**<.0001**
Male, % (n)	73 (8)	57 (12)	78 (40)	67 (51)	.70	.23
Weight, kg	78 [72, 90]	80 [73.4, 95.8]	90 [78, 103]	77.1 [69.7, 91.0]	.08	**.0008**
Height, m	1.7 [1.6, 1.7]	1.7 [1.6, 1.8]	1.8 [1.7, 1.8]	1.7 [1.6, 1.8]	**.0001**	**.0002**
Hypertension, % (n)	36 (4)	24 (5)	41 (21)	67 (51)	1.00	**.006**
Dyslipidemia, % (n)	9 (1)	5 (1)	20 (10)	24 (18)	.67	.67
Diabetes, % (n)	9 (1)	5 (1)	6 (3)	4 (3)	.55	.68
Smoking, % (n)	73 (8)	48 (10)	39 (20)	45 (34)	.052	.59
HTAD, % (n)	0 (0)	0 (0)	20 (10)	4 (3)	.19	**.006**
BAV, % (n)	0 (0)	0 (0)	35 (18)	46 (35)	**.02**	.27
AI, % (n)	0 (0)	0 (0)	41 (21)	43 (33)	**.01**	.86
AS, % (n)	0 (0)	0 (0)	8 (4)	29 (22)	1.00	.004
Diameter, mm	30.5 [25.5, 36.3]	28 [27, 34]	50.0 [46.0, 54.0]	51 [46.3, 57.8]	**<.0001**	.22

Statistically significant values are bolded.

*HTAD*, Hereditary thoracic aortic disease; *BAV*, bicuspid aortic valve; *AI*, moderate or more aortic insufficiency; *AS*, moderate or more aortic stenosis.

**Figure 2:**
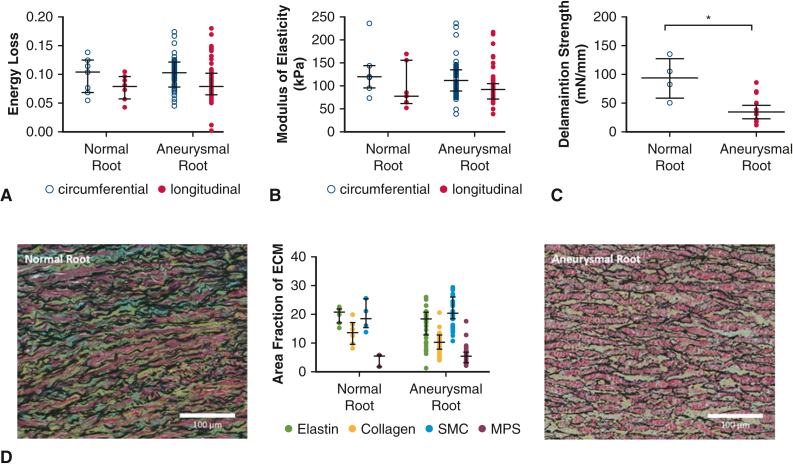
Biomechanical comparison between normal aortic roots and aneurysmal aortic roots. Normal aortic roots demonstrated similar energy loss (A) and modulus of elasticity (B) to aneurysmal aortic roots. Normal aortic roots exhibited greater delamination strength compared with aneurysmal aortic roots (C). All data are displayed as median with interquartile range. (D) Percent area of components of the aortic media were similar between normal aneurysmal aortic roots. ∗*P* ≤ .05. *SMC*, Smooth muscle cell; *MPS*, mucopolysaccharide.

Energy loss and modulus of elasticity were similar whether the aortic root was normal or aneurysmal. However, the delamination strength was greater for normal aortic roots than aneurysmal aortic roots (Figure 2).

On histologic analysis, percent area of elastin (*P* = .18), collagen (*P* = .11), smooth muscle cells (*P* = .50), and mucopolysaccharides (*P* = .33) were comparable between normal and aneurysmal aortic roots (Figure 2, *D*).

### Comparing Normal and Aneurysmal Ascending Aorta

Patients with aneurysms of the ascending aorta were older than their normal controls (67 [57, 75] years vs 46 [35, 55] years, *P* < .001), and had greater rates of hypertension. Nearly one half of the patients with aneurysms had bicuspid aortic valves (35/76, 46%) and significant rates of aortic valve dysfunction were present (Table 1).

**Figure 3:**
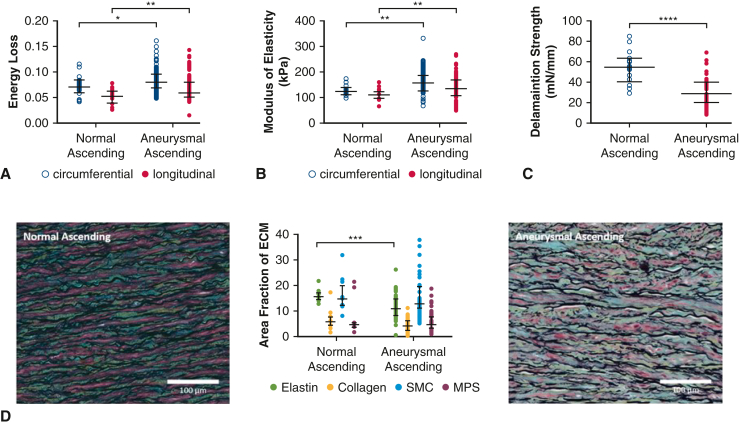
Biomechanical comparison between normal ascending aortas and aneurysmal ascending aortas. Aneurysmal ascending aortas exhibited greater energy loss than normal in both circumferential and longitudinal axes (A). Similarly, modulus of elasticity was also increased in aneurysmal ascending aortas (B). Normal ascending aortas exhibited greater delamination strength in comparison with aneurysmal ascending aortas (C). All data are displayed as median with interquartile range. Percent area of elastin was significantly greater in normal controls compared with aneurysms of the ascending aorta. Histologic comparison between normal and aneurysmal ascending aortas (D). Percentage area of elastin was significantly greater in normal ascending aortas compared with aneurysmal ascending aortas. ∗*P* ≤ .05, ∗∗*P* ≤ .01, ∗∗∗*P* ≤ .001, ∗∗∗∗*P* ≤ .0001. *SMC*, Smooth muscle cell; *MPS*, mucopolysaccharide.

Aneurysmal ascending aortas demonstrated greater energy loss and modulus of elasticity relative to normal controls. Delamination strength was lower for aneurysms than normal ascending aortas (Figure 3).

On histologic analysis, percent area of elastin was significantly greater in normal controls compared with aneurysms of the ascending aorta (*P* < .001) (Figure 3, *D*). The other aortic wall components analyzed were similar.

### Comparing Aneurysmal Ascending and Root Tissue

Patients with aneurysms of the ascending aorta were older (67 [57, 75] years vs 53 [39, 62] years, *P* < .0001) and with greater rates of hypertension compared with patients with aortic root aneurysms (67% [51/76] vs 41% [21/51], *P* = .006). However, the maximal diameters were similar between these 2 segments of aorta (51 [46, 58] mm vs 51 [47, 54] mm, *P* = .35) (Table 1).

**Figure 4:**
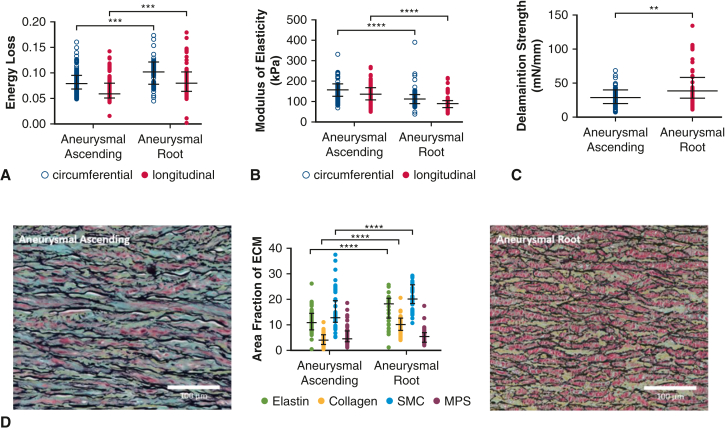
Biomechanical comparison between aneurysmal ascending aortas and aneurysmal aortic roots. Aneurysmal aortic roots exhibited greater energy loss (A), and lower modulus of elasticity (B) than aneurysmal ascending aortas in both the circumferential and longitudinal axis of aortic tissue. Aneurysmal aortic roots also exhibited greater delamination strength than aneurysmal ascending aortas (C). (D) Percent area of elastin, collagen, and smooth muscle cells were greater in aortic root aneurysms. All data are displayed as median with interquartile range. ∗∗*P* ≤ .01, ∗∗∗*P* ≤ .001, ∗∗∗∗*P* ≤ .0001. *SMC*, Smooth muscle cell; *MPS*, mucopolysaccharide.

The aortic root group demonstrated greater levels of energy loss (circumferential: *P* = .001; longitudinal: *P* < .001) and lower levels of modulus of elasticity (circumferential: 110.5 [86.9, 134.2] vs 156.5 [125.6, 185.3], *P* < .0001; longitudinal: 90.5 [71.0, 104.3] vs 134.3 [107.1, 167.6], *P* < .0001) (Figure 4). Aortic root aneurysms had greater delamination strength (*P* = .003). On histologic analysis, percent area of elastin, collagen and smooth muscle cells were greater in aortic root aneurysms (Figure 4, *D*).

**Figure 5:**
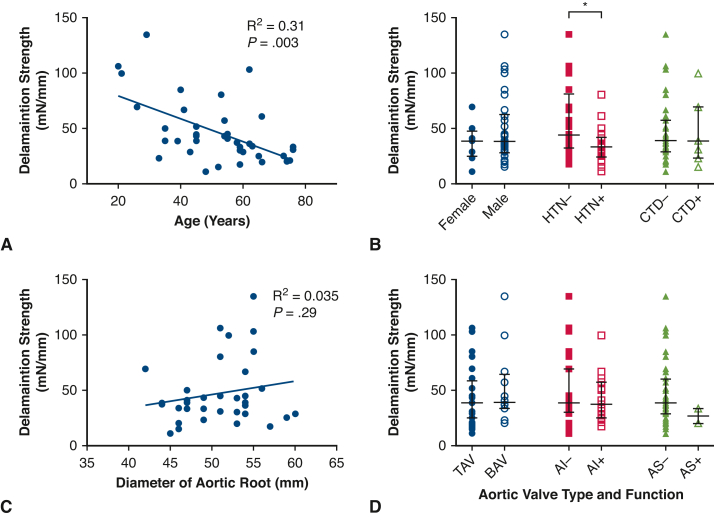
The impact of baseline characteristics on delamination strength in the aortic root. Increased age is associated with decreasing delamination strength (A). Delamination strength decreases with presence of hypertension but not with presence of connective tissue disorder, or difference in sex (B). Size of the aortic root was not associated with changes in delamination strength (C). Aortic valve type and function had no impact on delamination strength (D). ∗*P* ≤ .05. *HTN*, Hypertension; *CTD*, connective tissue disease; *TAV*, tricuspid aortic valve; *BAV*, bicuspid aortic valve; *AI*, moderate or more aortic insufficiency; *AS*, moderate or more aortic stenosis.

Using linear regression and *t* test, we examined the association of age, sex, hypertension, presence of known hereditary aortic disorder, aortic valve morphology, aortic valve function, and aortic size with delamination strength in the aortic root. Increased age and the presence of hypertension were strongly associated with decreased delamination strength in the aortic root (Figure 5). No other baseline characteristics were associated with delamination strength, including aortic size (*P* = .29).

To complement the regression analysis, propensity score matching was used to generate 23 well-matched pairs of patients with ascending aortic and aortic root aneurysms. After propensity score matching, baseline characteristics including age, hypertension, and aortic size were comparable, and the differences in energy loss and modulus of elasticity persisted (Table 2). Energy loss was greater in the aortic root than the ascending aorta in normal controls. Furthermore, modulus of elasticity was increased in aneurysmal ascending aorta compared with normal controls.

**Table 2:** Biomechanical differences between propensity-matched pairs of aneurysmal ascending and root patients

Biomechanical parameters	Matched aneurysmal ascending N = 23	Matched aneurysmal root N = 23	*P* value
Energy loss – Circ	0.077 [0.068, 0.091]	0.112 [0.085, 0.119]	.001
Energy loss – Long	0.054 [0.048, 0.065]	0.075 [0.061, 0.090]	.006
Modulus of elasticity – Circ, kPa	179.0 [155.8, 190.4]	115.4 [101.6, 137.4]	<.001
Modulus of elasticity – Long, kPa	159.7 [130.6, 173.1]	95.9 [79.2, 108.9]	<.001
Delamination strength, mN/mm	30.5 [20.0, 40.1]	33.9 [21.1, 42.0]	.63

*Circ*, Circumferential; *Long*, longitudinal.

Among the 23 propensity score–matched pairs of ascending and root aneurysms, no difference in delamination strength was observed (ascending: 30.5 [20.0, 40.1] mN/mm, root: 33.9 [21.1, 42.0] mN/mm, *P* = .63). However, given that normal ascending and root tissue differed in baseline delamination strength, we calculated change in delamination strength from baseline normal values. We then found that when baseline characteristics were controlled for, aneurysmal aortic roots demonstrated a greater change from normal in terms of delamination strength (Δ −59 mN/mm) than aneurysmal ascending aortas (Δ −24 mN/mm).

## Discussion

**Figure 6:**
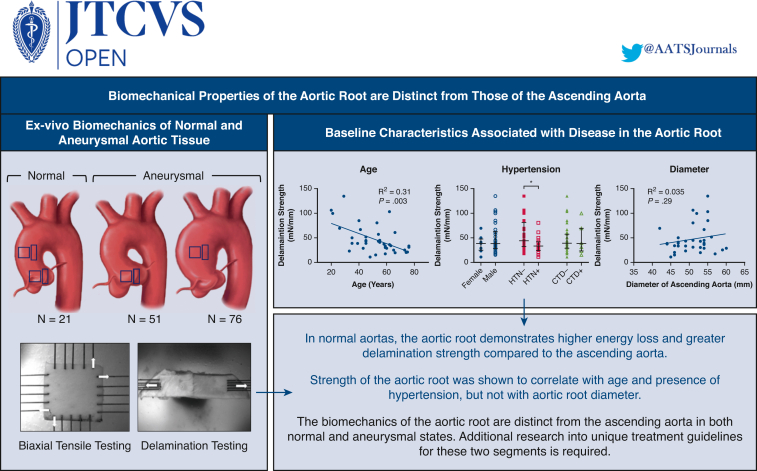
Biomechanical differences between the ascending aorta and aortic root considering normal and aneurysmal states. Healthy ascending and root tissue from 21 transplant donors (root = 11, ascending = 21), root tissue from 51 aneurysmal root patients, and ascending tissue from 76 aneurysmal ascending patients were collected and subjected to mechanical testing. The normal aortic root demonstrated greater resistance against delamination of the layers of the aortic tissue compared with the normal ascending aortic tissue. Similar results were found when comparing the aneurysmal ascending and root tissue. *HTN*, Hypertension; *CTD*, connective tissue disease.

In a detailed biomechanical analysis of ascending aortic and aortic root tissue, we characterized important differences in how these 2 segments of the aorta behave in both the normal state as well as aneurysmal states. The normal aortic root demonstrated greater viscoelastic behavior (greater energy loss or decreased ability to perform the Windkessel function) compared with normal ascending aortas but also greater resistance against delamination of the layers of the aortic wall as occurs in aortic dissection. In their aneurysmal states, both the root and the ascending aorta demonstrated decreased delamination strength compared with their normal states. However, unlike in the ascending aorta where increases in energy loss and tangent modulus of elasticity occur when aneurysms form, these properties were relatively preserved for aortic root aneurysms. Therefore, while we had previously been able to correlate energy loss with delamination strength in the ascending aorta, we were unable to do this for the aortic root. After matching for baseline characteristics, aneurysmal aortic roots had similar delamination strength to their ascending counterparts, but this was difficult to interpret, as normal delamination strength was different for these 2 different segments of aorta. These findings suggest that mechanical parameters need to be recalibrated for the aortic root and that novel mechanical parameters associated with decrease in strength are needed for the root (Figure 6).

Previous work on characterizing the biomechanical properties of the aortic root have focused on normal human and porcine aortic roots.^16^, ^17^, ^18^, ^19^ Significant differences exist between the mechanical properties of human versus porcine roots, limiting the interpretation of porcine data.^20^ Focusing on human studies, there are conflicting data on the differences in tissue stiffness between the aortic root and the ascending aorta, highlighting the limitations of stiffness in describing the nonlinear stress-strain behavior of aortic tissue.^19^, ^20^, ^21^ Xuan and colleagues^21^ evaluated failure stresses of 19 normal human aorta and found no difference between values in the ascending aorta and the aortic root, although interestingly, the lowest level of failure stress was located around the sinotubular junction between these 2 regions. By broadening the biomechanical parameters under investigation, we were able to identify additional important differences in energy loss and delamination strength between the 2 regions.

Another major finding of this study was the identification of aging and the presence of hypertension as associated with weakening of the aortic root, while diameter did not have this association. Iliopoulos and colleagues^22^ conducted one of the few reported biomechanical analyses of the aneurysmal aortic root. Aortic roots were excised from 13 patients during elective surgery. They found significant dependence of peak elastic modulus and failure stretch and stress on age, and the absence of associations between these biomechanical parameters and aortic root diameter. We corroborated the findings of biomechanical deterioration in the root with age and not with diameter in our larger dataset and found additionally the importance of hypertension. In a follow-up study by the same group, they included control specimens and found that directional dependency of aortic root aneurysms was lost.^23^ In our larger dataset, we continued to observe directional dependency in aneurysmal tissue, albeit at an attenuated level. We have also previously described this relative loss of directional dependency in aneurysmal ascending aorta.^12^ Any future multivariable predictive tool for aortic root aneurysm risk should incorporate age and hypertension, and possibly loss of directional dependency.

Although diameter was not associated with biomechanical properties, this commonly used variable alters geometry and may therefore affect wall stress. The Tseng group has extensive experience with finite element modeling of the aorta and recently reported findings in 46 patients with Marfan syndrome and aortic root aneurysms.^18^ In this relatively large series, they found that diameter did not correlate with wall stresses and advocated for patient specific modeling.^18^ Unfortunately, this is challenging in everyday clinical settings.

The next steps for improving risk prediction in the aortic root should move beyond diameter, but in addition to the incorporation of age and hypertension into prediction models, even more precise tools are necessary. Detailed studies correlating genetic and proteomic profiles with aortic biomechanics will be key and this is the subject of our group's ongoing work. Although the present analysis did not find an association with delamination strength and Marfan syndrome or other hereditary aortic disorders, such dichotomization of genetic disorders may have been too crude an analysis. Furthermore, we will continue to explore aortic root-specific biomechanical markers for aortic wall weakening.

There are limitations to biomechanical analyses. Delamination strength is a surrogate end point for resistance to aortic dissection. Although imperfect, we have previously demonstrated that delamination strength is significantly greater in those with normal ascending aortas relative to those who have suffered acute type A aortic dissection, and those with nondissected aortic aneurysms had delamination strength in between these 2 extremes. There are also minimum tissue sizes that we require to carry out the experiments. Consequently, only the noncoronary sinus was sampled from for the aortic root, although there is evidence that the 3 sinuses behave similarly.^19^ Finally, although our study is quite large compared with previous work, a greater number of patients and matched pairs would improve our confidence in adjusting for confounders.

## Conclusions

The normal aortic root was found to have distinct biomechanical properties compared with the normal ascending aorta. When aneurysms form in the aortic root, there is less strength against delamination compared with normal roots. This is similar to findings observed between aneurysmal and normal ascending aortas. However, other biomechanical changes such as increased energy loss are not observed between aneurysmal and normal aortic root, contrasting what is observed in the ascending aorta. Age and hypertension were associated with decreased aortic wall strength in the aortic root, while diameter had no such association.

## Conflict of Interest Statement

The authors reported no conflicts of interest.

The *Journal* policy requires editors and reviewers to disclose conflicts of interest and to decline handling or reviewing manuscripts for which they may have a conflict of interest. The editors and reviewers of this article have no conflicts of interest.
